# Balancing the Number of Quantum Wells in HgCdTe/CdHgTe Heterostructures for Mid-Infrared Lasing

**DOI:** 10.3390/nano12244398

**Published:** 2022-12-09

**Authors:** Mikhail A. Fadeev, Alexander A. Dubinov, Anna A. Razova, Arina A. Yantser, Vladimir V. Utochkin, Vladimir V. Rumyantsev, Vladimir Ya. Aleshkin, Vladimir I. Gavrilenko, Nikolai N. Mikhailov, Sergey A. Dvoretsky, Sergey V. Morozov

**Affiliations:** 1 Institute for Physics of Microstructures of RAS, 603950 Nizhny Novgorod, Russia; 2 Faculty of Radiophysics, Lobachevsky State University, 603950 Nizhny Novgorod, Russia; 3 Faculty of Physics, Lobachevsky State University, 603950 Nizhny Novgorod, Russia; 4 Advanced School of General and Applied Physics, Lobachevsky State University, 603950 Nizhny Novgorod, Russia; 5 Rzhanov Institute of Semiconductor Physics, Siberian Branch, Russian Academy of Sciences, 630090 Novosibirsk, Russia

**Keywords:** HgCdTe, optical gain, stimulated emission, mid-IR

## Abstract

HgCdTe-based heterostructures with quantum wells (QWs) are a promising material for semiconductor lasers in the atmospheric transparency window (3–5 μm) thanks to the possibility of suppressing Auger recombination due to the no-parabolic law of carrier dispersion. In this work, we analyze the thresholds of stimulated emission (SE) under optical pumping from heterostructures with a different number of QWs in the active region of the structure. Total losses in structures are determined from the comparison of thresholds for the different number of QWs in the active region. It is shown that, thanks to the increased modal gain, a higher number of QWs results in lower threshold pumping intensity and, consequently, higher temperature of SE. These results indicate that improvements to the modal gain can result in a moderate uplift in the temperature of SE from mid-infrared HgCdTe-based heterostructures. On the other hand, at a high enough QW count threshold, the intensity no longer depends on the number of the QWs and is determined by the transparency concentration of a single QW.

## 1. Introduction

Coherent sources of radiation for mid-infrared (IR) spectral range are in demand for a variety of spectroscopic and other applications. They can be utilized in non-invasive medical diagnostics, industrial process control, environmental monitoring, atmospheric sensing and free space communication. In regard to environment monitoring, particular interest is attracted to devices which operate in the atmospheric transparency window from 3 to 5 μm, as this range covers the absorption lines of common pollutants and greenhouse gases [1].

Despite the prevalence of interband semiconductor diodes in visible and near-IR ranges, their operation in the mid-IR range until recently has been limited to either cryogenic temperatures or short wavelengths. The main obstacle to develop mid-IR semiconductor lasers is Auger recombination (AR), a non-radiative process the rate of which increases with temperature and wavelength of emission. AR reduces carrier lifetime and leads to the heating of non-equilibrium carriers, which drastically increases the threshold of laser generation.

There are several ways to reduce the negative effects of AR. The use of intraband transitions is the main principle behind quantum cascade lasers (QCLs). Mid-IR QCLs, which were enabled by the advances in semiconductor growth and processing technology, provide exceptional figures of merit in the range from 4 to 15 μm [2,3,4]. At the same time, mass production of QCLs is limited by the complexity of their design. A different approach consists in using interband transitions and engineering the band structure of the active region of the laser in order to suppress AR. It can be realized using III–V (GaSb, InAs) heterostructures with either type-I or type-II quantum wells (QWs) [5]. Diode lasers based on III–V materials were shown to operate in mid-IR range at room temperature but yet are limited to the shortwave region of 3–5 μm window. For lasers with type-I QWs, room-temperature high-power lasing is achieved up to 3.3 μm [6]. However, extending the wavelength beyond 3 μm requires increasingly complex structure designs with maximum room temperature emission currently being 3.7 μm for structures with quinternary barriers [7]. For type-II QWs, lasing at 4 μm wavelength (at T = 308 K) was reported in [8] (though lasing threshold exceeded 20 kW/cm^2^, compared to typically less than 1 kA/cm^2^ threshold current for shorter-wavelength, type-I QW lasers). Following this path, the best results were achieved in interband cascade lasers, which incorporate the cascade principle of QCLs and an active region designed to reduce AR [9,10]. Combination of these factors leads to remarkable performance comparable to QCLs in the range up to 6 μm but also gives interband cascade lasers the drawbacks of QCLs, such as the complexity of the design. An alternative solution is to use semiconductor materials (II-VI or IV-VI), for which Auger recombination is naturally suppressed. In this regard, lead chalcogenide lasers are a perfect example, as mirror-like dispersion law enabled their operation in the whole mid-IR range up to 50 µm [11,12] despite technological difficulties, which complicate the formation of heterostructures. HgCdTe is another interesting material system, for which molecular beam epitaxy provides high-quality HgTe/CdHgTe heterostructures and although bulk HgCdTe layers demonstrate Auger coefficients higher than in III–V lasers [13], the use of Hg(Cd)Te/CdHgTe QWs is shown to mitigate AR [14,15]. It should be mentioned, that while mitigating bulk-like AR, introduction of heterointerfaces may enable two types of QW-specific Auger processes. During the first, one electron or hole migrates to a higher quantization sub-band, during the other it migrates to the continuum of barrier layers [16]. These processes are generally prohibited in long-wavelength Hg(Cd)Te/CdHgTe structures, as the energy of interband transition is not high enough to excite the electron to a higher level. For structures which emit in the 3–5 μm region, these processes are possible; however, they typically do not manifest themselves until temperatures above 200 K [15]. Moreover, as suggested in [17], maximizing the band offsets by utilizing Cd-rich (Cd_x_Hg_1−x_Te, x > 0.9) barrier layers can further increase this temperature. Recently, stimulated emission (SE) was obtained from HgCdTe-based heterostructures up to 240 K at the wavelength of 3.7 μm [15] and at room temperature at the wavelength of 2.7 μm [18], while theory predicts the possibility of 3 μm diode lasers operating at room temperature [19].

Although HgCdTe-based structures cannot yet operate at room temperature in the 3–5 μm range, significant improvements over recent years suggest that it can become feasible for better optimized structures. Modification of the active region of the laser in order to suppress AR is the main way to increase the temperature and the wavelength of laser operation. At the same time, certain improvements can be achieved without changing the parameters of the QWs, by increasing the modal gain of the structure. The simplest way to increase the modal gain is to increase the number of the QWs in the active region, which leads to almost linear growth of the confinement factor and, consequently, the modal gain. In this work, we investigate the prospects of increasing the operating temperature of HgCdTe-based lasers by changing the number of the QWs in the active region. We compare the characteristics of SE from structures with a different number of the QWs and estimate the necessary increment to this number to push the emission to higher temperatures.

## 2. Materials and Methods

Structures studied in this work were grown by molecular beam epitaxy on semi-insulating GaAs (013) substrates with subsequent with in-situ ellipsometric control of the layer content and thickness [20]. Oxide desorption from GaAs substrate was performed in As-rich environment at ~580 °C. Subsequent ZnTe and CdTe buffers were grown at ~280–300 °C and ~285–295 °C, correspondingly [21], which was followed by waveguide layers and QWs grown at 180–190 °C [22]. All structures have a 650 nanometer-wide Hg_0.25_Cd_0.75_Te waveguide layer which serves for optical confinement of the TE_0_ mode near the active core of the structure. The active core consists of multiple Hg_x−1_Cd_x_Te QWs separated by 30 nm Hg_0.25_Cd_0.75_Te barriers. In this work, we studied three heterostructures which differed in the number of QWs in the active core (3, 5 and 10 2.7 nanometer-wide QWs, which corresponds to optical confinement factors of Γ_3_ = 0.004, Γ_5_ = 0.0083, Γ_10_ = 0.016).

**Figure 1:**
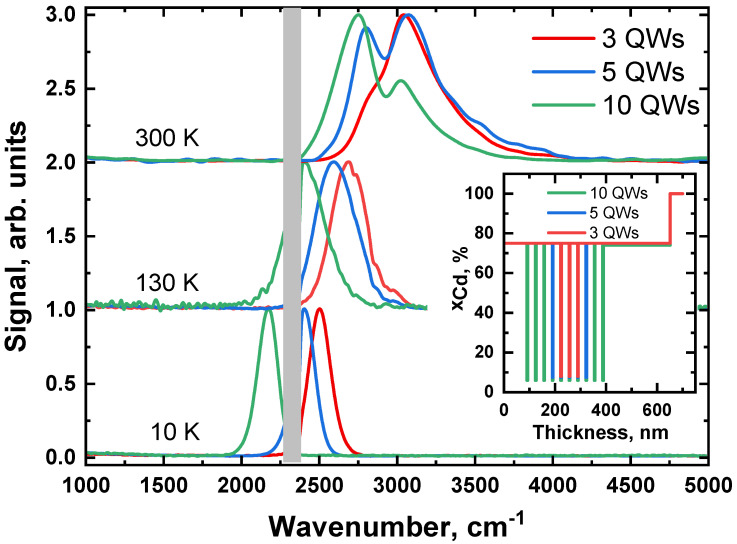
Photoluminescence spectra of Hg_1−x_Cd_x_Te quantum well (QW) structures with different number of QWs at different temperatures under 808 nm diode laser excitation; grey box covers the region of high atmospheric absorption at ≅2330 cm^−1^. The inset shows the growth scheme of the samples, zero correspond to the end of 10-μm CdTe buffer.

Ex situ characterization was performed using temperature-dependent photoluminescence (PL) spectroscopy [23]. The sample was mounted on a cold finger of helium closed-cycle refrigerator and optically pumped with an 808 nm diode laser; PL signal was collected from top of the structure. Spectra were recorded using Bruker Vertex 80v Fourier spectrometer. PL spectra of the studied structures are shown in Figure 1. PL lines of structures with 3 and 5 QWs have a wavelength of ~4.1 μm at 10 K, while the structure with 10 QWs has a longer wavelength of ~4.6 μm. As the temperature grows, all PL lines shift to shorter wavelengths with the rate of 2 cm^−1^K^−1^, typical for Hg(Cd)Te/HgCdTe QWs. At 300 K, the peaks of PL lines lie at 3.3 μm, 3.5 μm and 3.6 μm for structures with 3, 5 and 10 QWs, correspondingly. Based on the position and temperature shift of the PL lines, the parameters of the QWs were determined. First, the bandgap of the structure was determined, then the parameters of the QWs were found by solving an inverse problem of computing the PL spectrum from the parameters of the QW for different temperatures. The difference in PL position indicates different CdTe content of QWs of different structures, which is ~6% for structure with 10 QWs and ~8% for structures with 3 and 5 QWs; however, this difference does not affect the value of the Auger threshold energy, which is 56 meV and 55 meV for structures with 10 and 5 QWs, correspondingly.

For the measurements of stimulated emission, the samples of 4 × 4 mm^2^ were cleaved from the wafer and placed on the cold finger of the closed-cycle cryostat. Optical pumping was provided by the pulsed parametric oscillator “Solar” (1.6 μm wavelength, 10 ns pulse duration, 10 Hz repetition rate, 5 mW—maximum average power; attenuation was achieved with a set of optical filters). Excitation was performed from the top of the structure and emission was collected from the cleaved facet of the sample and guided to the Fourier spectrometer equipped with an HgCdTe-based detector. Photon energy in the experiment was smaller than the bandgap of the barrier layers, thus non-equilibrium carriers were excited in each QW independently. Details of the experimental method and the scheme of the experimental set-up can be found in Supplementary Materials (Figure S1).

## 3. Results

**Figure 2:**
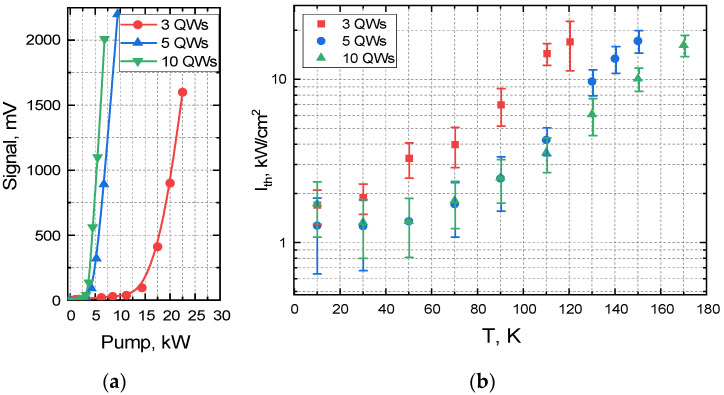
(**a**) Dependence of photoluminescence signal on pumping power at T = 110 K, (**b**) Threshold pumping intensity (I_th_) under pulsed pumping (1.5 µm wavelength, 10 ns pulse duration, 10 Hz repetition rate) for different temperatures (T). 3, 5, 10 QWs—number of quantum wells in the structure.

The temperature dependencies of the threshold pumping intensity for the studied structures are shown in Figure 2b. Threshold pumping intensity was determined from the measurements of signal vs. pumping dependencies (Figure 2a) by linear extrapolation of the curve to zero signal. Full data and an example of data treatment can be found in Supplementary Materials (Figures S2 and S3). Obtained values were verified by recording spectra in the vicinity of threshold intensity and controlling the decrease of the width at half maximum of the emission spectra.

At cryogenic temperatures, the thresholds for structures with three and five QWs are practically the same, but as the temperature grows, their difference increases both in absolute and relative value. When comparing the structures with 10 and 5 QWs, the trend is similar: in the temperature range up to 160 K the thresholds remain almost equal, while at higher temperatures the difference becomes larger.

Threshold pumping intensity, which is necessary for the onset of stimulated emission in heterostructure with the number of the QWs, N_QW_, is described by the following equation:

(1) $$I_{n}=\hbar {\Omega R}_{\mathrm{th}}/\eta ,$$

where ℏΩ and η are the energy of the photon and the portion of the absorbed pumping photons correspondingly; R_th_ is the recombination rate of non-equilibrium carriers. Equation (1) is conditioned by the assumptions that pumping radiation is absorbed only in the QWs and overall absorption is low ($\hbar \Omega <E_{g}$ and Nη≪1, where E_g_ is the bandgap of the waveguide and barrier layers). The recombination rate of non-equilibrium carriers is described within the ABC model:

(2) $$R_{\mathrm{th}}=\mathrm{An}+{\mathrm{Bn}}^{2}+{\mathrm{Cn}}^{3},$$

where A, B and C are the coefficients of Shockley–Read–Hall, radiative and Auger recombination in the QW, correspondingly; n is the threshold carrier concentration. The ratio of threshold pumping intensities for structures with similar parameters but with different number (i and j) QWs in the active region is determined only by the values of threshold carrier concentrations (n_i,j_) for each of the structures:

(3) $$\frac{I_{i}}{I_{j}}=\frac{{\mathrm{An}}_{i}+{\mathrm{Bn}}_{i}^{2}+{\mathrm{Cn}}_{i}^{3}}{{\mathrm{An}}_{j}+{\mathrm{Bn}}_{j}^{2}+{\mathrm{Cn}}_{j}^{3}}.$$

**Figure 3:**
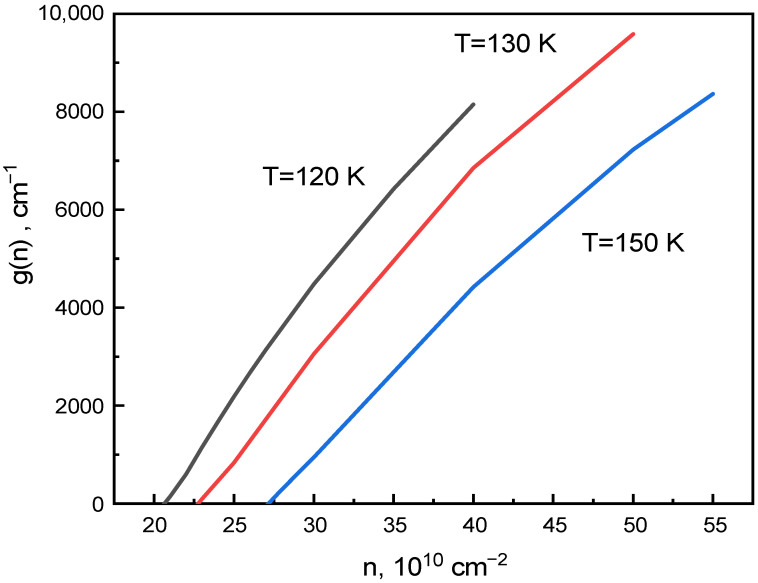
Calculated dependence of g(n) for 2.7 nm wide Hg_0.23_Cd_0.77_Te/Hg_0.1_Cd_0.9_Te/Hg_0.23_Cd_0.77_Te QW for three temperatures: 120 K, 130 K, 150 K.

The threshold carrier concentration can be found from the condition of the onset of laser generation:

(4) $$g\left(n_{i}\right)\Gamma_{i}=\alpha ,$$

where α a is a total loss coefficient in the structure (including radiation losses), Γ_i_ is optical confinement factor for structure with i QWs, and g(n_i_) is the material amplification coefficient for one QW. Following the calculations from [24], it can be shown that the material gain coefficient in Hg_x_Cd_1−x_Te/Hg_y_Cd_1−y_Te QW in a wide range of carrier concentrations can be expressed as:

(5) $$g\left(n\right)=G\cdot \left(n-n^{\mathrm{tr}}\right),$$

where G is a coefficient which depends on the content, the width of the QW and the temperature, and $n^{\mathrm{tr}}$ is the transparency concentration which corresponds to $g(n^{\mathrm{tr}})=0$. The calculated gain coefficient for T = 120 K, 130 K and 150 K, shown in Figure 3. To calculate these values, we used Kane’s model, taking into account the effects of the embedded strain of the layers. For simplicity, we neglected the effects of symmetry lowering at heterojunctions and the absence of inversion center in the structure, which led to a twofold degeneration of the electronic spectrum. Details of the calculations can be found in the paper [24]. Taking into account Expressions (4) and (5), Expression (3) can be written as:

(6) $$\frac{I_{i}}{I_{j}}=\frac{A\left(n^{\mathrm{tr}}+\frac{\alpha }{{G\Gamma }_{i}}\right)+B{\left(n^{\mathrm{tr}}+\frac{\alpha }{{G\Gamma }_{i}}\right)}^{2}+C{\left(n^{\mathrm{tr}}+\frac{\alpha }{{G\Gamma }_{i}}\right)}^{3}}{A\left(n^{\mathrm{tr}}+\frac{\alpha }{{G\Gamma }_{j}}\right)+B{\left(n^{\mathrm{tr}}+\frac{\alpha }{{G\Gamma }_{j}}\right)}^{2}+C{\left(n^{\mathrm{tr}}+\frac{\alpha }{{G\Gamma }_{j}}\right)}^{3}}.$$

## 4. Discussion

Expression (6) is based on general assumptions of a linear amplification regime and low total absorption of pumping radiation and thus can be applied to experimental results in the whole temperature range. However, it is useful to consider special cases for which Expression (6) can be simplified. First, when the additional concentration necessary to overcome the losses in the structure is much smaller than the transparency concentration for one QW $\left(\alpha /{G\Gamma }_{i,j}\ll n^{\mathrm{tr}}\right)$, the threshold pumping intensity does not depend on the number of QWs in the structure. This situation is facilitated by low temperature of the sample, as low temperature results in low losses and high gain.

As the temperature increases, it becomes important which recombination mechanism is dominating at the transparency concentration, n^tr^. Depending on that, the ratio between threshold intensities is approximately equal to:

(7) $$\frac{I_{i}}{I_{j}}≅{\left(\frac{n^{\mathrm{tr}}+\frac{\alpha }{{G\Gamma }_{i}}}{n^{\mathrm{tr}}+\frac{\alpha }{{G\Gamma }_{j}}}\right)}^{q},$$

where q = 1, 2, 3 for pure SRH, radiative and Auger recombination, correspondingly. Expression (7) does not depend on the exact value of recombination rate of non-equilibrium carriers. In the case of high losses in structure: $\alpha /{G\Gamma }_{i,j}\gg n^{\mathrm{tr}}$ it simplifies to $I_{i}/I_{j}≅{\left(\Gamma_{j}/\Gamma_{i}\right)}^{q}$. When transparency concentration and additional concentration necessary to overcome the losses are comparable, the ratio lies between these values: $1<I_{i}/I_{j}<{\left(\Gamma_{j}/\Gamma_{i}\right)}^{q}\;$ for i < j.

Although AR is suppressed in HgTe/HgCdTe heterostructures, it still becomes dominating recombination mechanism at high enough temperature due to exponential growth of its rate. Using the value of Auger coefficient from [15] (C = 2.8 × 10^–15^ cm^4^/s, which corresponds to recombination time ~6 ns) and radiative recombination time (~20 ns) calculated analogously to [24], AR can be assumed to be dominating recombination mechanism at temperatures above 120 K.

Experimental values of the ratio of threshold intensities are given in Table 1. One can see that threshold intensities in structures with a different number of the QWs stay practically equal up to 30 K, which suggests that losses are low compared to the gain in all three structures. As the temperature increases, the ratio between thresholds for structures with 3 and 5 QWs starts growing. For a structure with three QWs, threshold intensity quickly becomes 2.5–4 times higher than for the structure with five QWs, which is indicative of the transition to the case of high losses; the largest ratio between thresholds for these structures is Γ_5_/Γ_3_ ≅ 4.6.

With the condition that AR is the main channel of interband recombination, we can extract the value of total loss coefficient from the experimental value of the threshold ratio, using the calculated values of G, n^tr^ and Γ_i_. Using the ratio between threshold intensities of structures with three and five QWs, the loss coefficient at 110 K is found to be 31.5 ± 9 cm^−1^. The same coefficient can be found from the ratio between the structures with five and ten QWs (Table 1). For these structures, the ratio between threshold intensities is supposed to be larger due to larger difference in the optical confinement factors, however, at 110 K $I_{5}/I_{10}≅1.2$, which corresponds to the loss coefficient of 0–26 cm^−1^. In total, we can obtain four independent values of α, all of which correspond to the range 20–40 cm^−1^, except for the outlier at 110 K, which can be a product of high error as well as the effect of radiative recombination, which leads underestimation of α.

**Figure 4:**
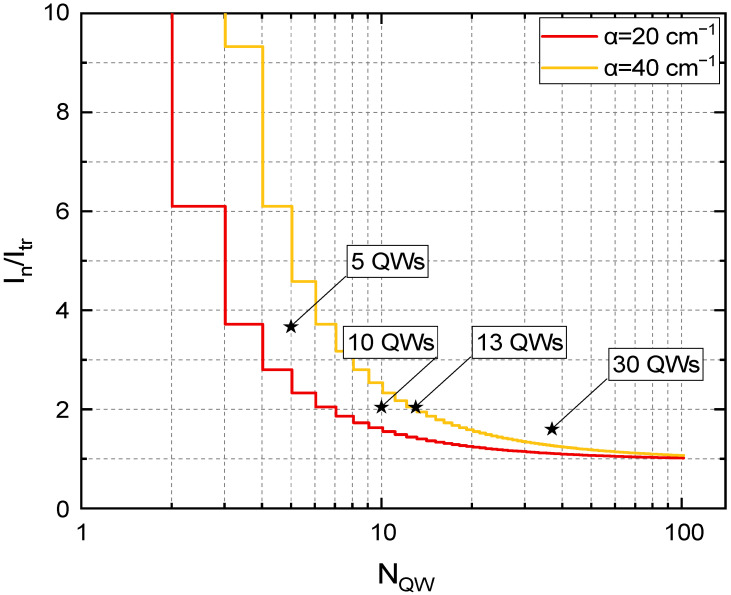
Threshold intensities for different number of quantum wells (N_QW_) in the active core divided by transparency intensity ($I_{\mathrm{tr}}=c{\left(n^{\mathrm{tr}}\right)}^{3}$) at T = 150 K. Solid lines: calculations for two values of absorption coefficient α, symbols: experimental values for structures with 5, 10, 13 and 30 quantum wells (QWs).

Figure 4 shows the dependence of threshold intensities at 150 K on the number of the QWs in the active core divided by the transparency intensity, $I_{\mathrm{tr}}=c{\left(n^{\mathrm{tr}}\right)}^{3}$. Two lines correspond to the boundaries of the loss coefficient range. As one can see, for the structure with 3 QWs, the threshold intensity is 5–9 times higher than the transparency intensity, while for the structure with 10 QWs, it is only twice as high as I_tr_ and this value weakly depends on the value of the loss coefficient. Star shapes in Figure 4 show the experimental results obtained for the structures with 5 and 10 QWs (this work), 13 QWs (Ref [15]) and 30 QWs (with optical confinement factor, Γ_30_ = 0.063, which is similar to the optimal structure with 37 QWs). These results show that when N_QW_ is low, additional QWs can provide multifold decrease of threshold pumping intensity, but the benefit of adding new QWs becomes insignificant when their number is high.

It should be noted that a simple model used in this work does not account for several effects. First, when the number of the QWs is above 20, it contradicts the assumption of low absorption of the pumping radiation and results in difference of non-equilibrium carrier concentrations in QWs located deeper in the structure and those closer to the surface, which would lead to higher thresholds than predicted by the model by effectively increasing the transparency intensity. Second, for too high number of the QWs it is difficult to place them in the antinode of the electric field, as the total thickness of 40 2.7 nm quantum wells separated by 10 nm barriers becomes comparable to the emission wavelength inside the structure. Third, this model does not consider thermal effects which can arise during high-duty cycle operation. At the fixed pumping intensity, more QWs in the active core would result in proportionally more generated heat, but the decrease in the threshold intensity with the number of QWs partially compensates for this effect, resulting in sublinear dependence. All of these effects are important when the number of QWs is high and all of them lead to higher threshold intensities than predicted in this work. However, the model we use adequately describes the results obtained for N_QW_ < 20 and it predicts that increasing N_QW_ above this value does not provide a significant decrease in threshold intensity, which is determined by the transparency concentration of a single QW.

The decrease in the threshold intensity can be translated into the increase in critical temperature of SE. As an example, we consider the structure with 10 QWs at 150 K. Its threshold intensity is approximately two times higher than the transparency intensity, which sets the lower limit of threshold intensity. Taking into account exponential growth of the threshold with a characteristic temperature of 35 K (see Figure 2), a twofold decrease in threshold would result in a 24 K improvement of critical temperature at the same power of optical pumping. For similar structure with 20 QWs, the maximum temperature improvement is 14 K. In order to increase the critical temperature by another 10 K, one would need to use 50 QWs, making structures hard to produce even from the technological point of view. Thus, we surmise that the sweet spot of the number of QWs in Hg(Cd)Te/HgCdTe heterostructures lies between 15 and 30.

## Figures and Tables

**Table 1:** The ratio between threshold pumping intensities of structures with 3 and 5 quantum wells (I_3_/I_5_), 5 and 10 quantum wells (I_5_/I_10_), calculated values of amplification (G) and transparency concentration (n^tr^) and loss coefficient (α) calculated using the value of I_5_/I_10_ for different temperatures (T).

T, K	I_3_/I_5_	I_5_/I_10_	G, nm	n^tr^_,_ cm^−2^	α, cm^−1^
10	1.3 ± 0.7	0.7 ± 0.4	--	--	--
30	1.5 ± 0.8	0.9 ± 0.6	--	--	--
50	2.4 ± 1.1	1 ± 0.6	--	--	--
70	2.3 ± 1	1 ± 0.5	--	--	--
90	2.8 ± 1.2	1 ± 0.5	--	--	--
110	3.4 ± 0.8	1.2 ± 0.45	0.49	2.3 * 10^11^	10 ± 16
130	--	1.6 ± 0.5	0.36	2.3 * 10^11^	22.5 ± 18
150	--	1.7 ± 0.4	0.32	2.9 * 10^11^	36.5 ± 9

## Data Availability

The data presented in this study are available on request from the corresponding author.

## Supplementary Materials

The following supporting information can be downloaded at: https://www.mdpi.com/article/10.3390/nano12244398/s1, Figure S1: Scheme of the experimental set-up; Figure S2: Dependence of PL signal intensity on pumping power at different temperatures for structures with: (a) 3 QWs; (b) 5 QWs; (c) 10 QWs; Figure S3: Pumping power versus stimulated emission intensity at different temperatures for structures with 3 QWs.

## References

[1] Gordon I.E., Rothman L.S., Hill C., Kochanov R.V., Tan Y., Bernath P.F., Birk M., Boudon V., Campargue A., Chance K.V. (2017). The HITRAN2016 Molecular Spectroscopic Database. J. Quant. Spectrosc. Radiat. Transf..

[2] Vitiello M.S., Scalari G., Williams B., De Natale P. (2015). Quantum Cascade Lasers: 20 Years of Challenges. Opt. Express.

[3] Bandyopadhyay N., Bai Y., Tsao S., Nida S., Slivken S., Razeghi M. (2012). Room Temperature Continuous Wave Operation of Λ~3–3.2 μm Quantum Cascade Lasers. Appl. Phys. Lett..

[4] Bandyopadhyay N., Bai Y., Slivken S., Razeghi M. (2014). High Power Operation of λ~5.2–11 Μm Strain Balanced Quantum Cascade Lasers Based on the Same Material Composition. Appl. Phys. Lett..

[5] Jung D., Bank S., Lee M.L., Wasserman D. (2017). Next-Generation Mid-Infrared Sources. J. Opt..

[6] Belenky G., Shterengas L., Kipshidze G., Hosoda T. (2011). Type-i Diode Lasers for Spectral Region above 3 Μm. IEEE J. Sel. Top. Quantum Electron..

[7] Vizbaras K., Amann M.C. (2012). Room-Temperature 3.73 Μm GaSb-Based Type-I Quantum-Well Lasers with Quinternary Barriers. Semicond. Sci. Technol..

[8] Veerabathran G.K., Sprengel S., Andrejew A., Amann M.C. (2017). Room-Temperature Vertical-Cavity Surface-Emitting Lasers at 4 Μm with GaSb-Based Type-II Quantum Wells. Appl. Phys. Lett..

[9] Meyer J.R., Bewley W.W., Canedy C.L., Kim C.S., Kim M., Merritt C.D., Vurgaftman I. (2020). The Interband Cascade Laser. Photonics.

[10] Yang R.Q. (2013). Interband Cascade (IC) Lasers.

[11] Preier H. (1979). Recent Advances in Lead-Chalcogenide Diode Lasers. Appl. Phys..

[12] Maremyanin K.V., Ikonnikov A.V., Bovkun L.S., Rumyantsev V.V., Chizhevskii E.G., Zasavitskii I.I., Gavrilenko V.I. (2018). Terahertz Injection Lasers Based on a PbSnSe Solid Solution with an Emission Wavelength up to 50 Μm and Their Application in the Magnetospectroscopy of Semiconductors. Semiconductors.

[13] Meyer J.R., Canedy C.L., Kim M., Kim C.S., Merritt C.D., Bewley W.W., Vurgaftman I. (2021). Comparison of Auger Coefficients in Type i and Type II Quantum Well Midwave Infrared Lasers. IEEE J. Quantum Electron..

[14] Mami F.Z., Kadri A., Mokdad N., Zitouni K. (2021). Nonparabolicity Effects on Carrier Lifetimes in Bulk Hg1-XCdxTe Alloys and Mid-Infrared 2D Hg1-XCdxTe/CdTe Single Quantum Well Lasers. Superlattices Microstruct..

[15] Kudryavtsev K.E., Rumyantsev V.V., Aleshkin V.Y., Dubinov A.A., Utochkin V.V., Fadeev M.A., Mikhailov N.N., Alymov G., Svintsov D., Gavrilenko V.I. (2020). Temperature Limitations for Stimulated Emission in 3–4 μm Range Due to Threshold and Non-Threshold Auger Recombination in HgTe/CdHgTe Quantum Wells. Appl. Phys. Lett..

[16] Zegrya G.G., Polkovnikov A.S. (1998). Mechanisms of Auger Recombination in Quantum Wells. J. Exp. Theor. Phys..

[17] Vurgaftman I., Meyer J. (1998). High-Temperature HgTe/CdTe Multiple-Quantum-Well Lasers. Opt. Express.

[18] Utochkin V.V., Kudryavtsev K.E., Dubinov A.A., Fadeev M.A., Rumyantsev V.V., Razova A.A., Andronov E.V., Aleshkin V.Y., Gavrilenko V.I., Mikhailov N.N. (2022). Stimulated Emission up to 2.75 Μm from HgCdTe/CdHgTe QW Structure at Room Temperature. Nanomaterials.

[19] Afonenko A.A., Ushakov D.V., Dubinov A.A., Aleshkin V.Y., Morozov S.V., Gavrilenko V.I. (2022). Hot Phonon Effects and Auger Recombination on 3 μ m Room Temperature Lasing in HgTe-Based Multiple Quantum Well Diodes. J. Appl. Phys..

[20] Mikhailov N.N., Smirnov R.N., Dvoretsky S.A., Sidorov Y.G., Shvets V.A., Spesivtsev E.V., Rykhlitski S.V. (2006). Growth of Hg1-XCdxTe Nanostructures by Molecular Beam Epitaxy with Ellipsometric Control. Int. J. Nanotechnol..

[21] Dvoretsky S.A., Mikhailov N.N., Ikusov D.G., Kartashev V.A., Kolesnikov A.V., Sabinina I.V., Sidorov Y.G., Shvets V.A., Nánai L., Samantara A., Fábián L., Ratha S. (2019). The Growth of CdTe Layer on GaAs Substrate by MBE.

[22] Dvoretsky S., Mikhailov N., Sidorov Y., Shvets V., Danilov S., Wittman B., Ganichev S. (2010). Growth of HgTe Quantum Wells for IR to THz Detectors. J. Electron. Mater..

[23] Rumyantsev V.V., Razova A.A., Bovkun L.S., Tatarskiy D.A., Mikhailovskii V.Y., Zholudev M.S., Ikonnikov A.V., Uaman-Svetikova T., Maremyanin K.V., Utochkin V.V. (2021). Optical Studies and Transmission Electron Microscopy of Hgcdte Quantum Well Heterostructures for Very Long Wavelength Lasers. Nanomaterials.

[24] Aleshkin V.Y., Dubinov A.A., Rumyantsev V.V., Fadeev M.A., Domnina O.L., Mikhailov N.N., Dvoretsky S.A., Teppe F., Gavrilenko V.I., Morozov S.V. (2018). Radiative Recombination in Narrow Gap HgTe/CdHgTe Quantum Well Heterostructures for Laser Applications. J. Phys. Condens. Matter..

