# Supplementary material for: Balancing the Number of Quantum Wells in HgCdTe/CdHgTe Heterostructures for Mid-Infrared Lasing
Source: Nanomaterials (Basel). 2022 Dec 9;12(24):4398. doi: 10.3390/nano12244398 (PMC9785783; doi:10.3390/nano12244398)
Supplement: Supplementary file 1 [file nanomaterials-12-04398-s001.zip › nanomaterials-2024656-supplementary.pdf]

nanomaterials

Supplementary Materials

Experimental Set-Up

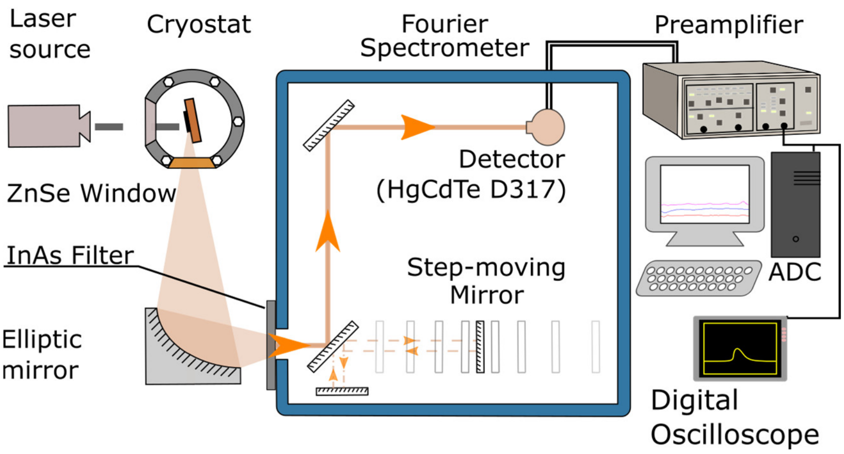

Figure S1. Scheme of the experimental set-up.

Stimulated emission requires higher excitation power which can be achieved by pulsed sources. In this work we used either OPO "Solar' with 10 Hz repetition rate and 10 ns pulse duration the wavelength of emission. Average power with a maximum of 5 mW was measured near the cryostat window and converted into peak power by dividing by the value of the duty cycle. Pumping radiation is directed to the sample by a set of mirrors. The sample was glued to the cold finger of the closed-cycle cryostat.; attenuation was achieved with a set of optical filters). Excitation was performed from top of the structure through quartz window and emission was collected from the cleaved facet of the sample and guided to the Fourier spectrometer through ZnSe window. The sample was put in the focal point of the elliptic mirror. The other focal point was aligned (focal point, direction and aperture) with the optical system of Fourier spectrometer Bruker Vertex 80v. For detection we used HgCdTe photovoltaic detector D317. The signal from the detector was passed through the SR560 preamplifier and split to digital oscilloscope which was user for integral PL measurement and ADC board which was used to record the spectra.

The pulsed nature of the SE does not allow using Lock-in amplifier for averaging the signal, thus it was directly passed to the ADC board after its amplification. The filters of the pre-amplifier were set to the narrowest range, which does not affect the shape of the signal. During all the measurements the Fourier spectrometer was operated in the step-scan mode, which enabled the measurements with low duty cycle. In this mode the moving mirror of the spectrometer is moving not at a constant speed, but in separate "steps", the length of which is determined by the required upper boundary frequency of the investigated spectrum, and the number - by spectral resolution. After the mirror is positioned, for each of the steps there is a delay, which allows the mirror to stabilize, before the actual measurements start.

Additional reduction of the background noise was achieved by utilizing time resolved PL spectroscopy. It is based on the fact that when SE starts, all the radiation is emitted over a short period of time (shorter than 10 ns). During that period the power of SE by far exceeds the background radiation while still being less intensive on the average.

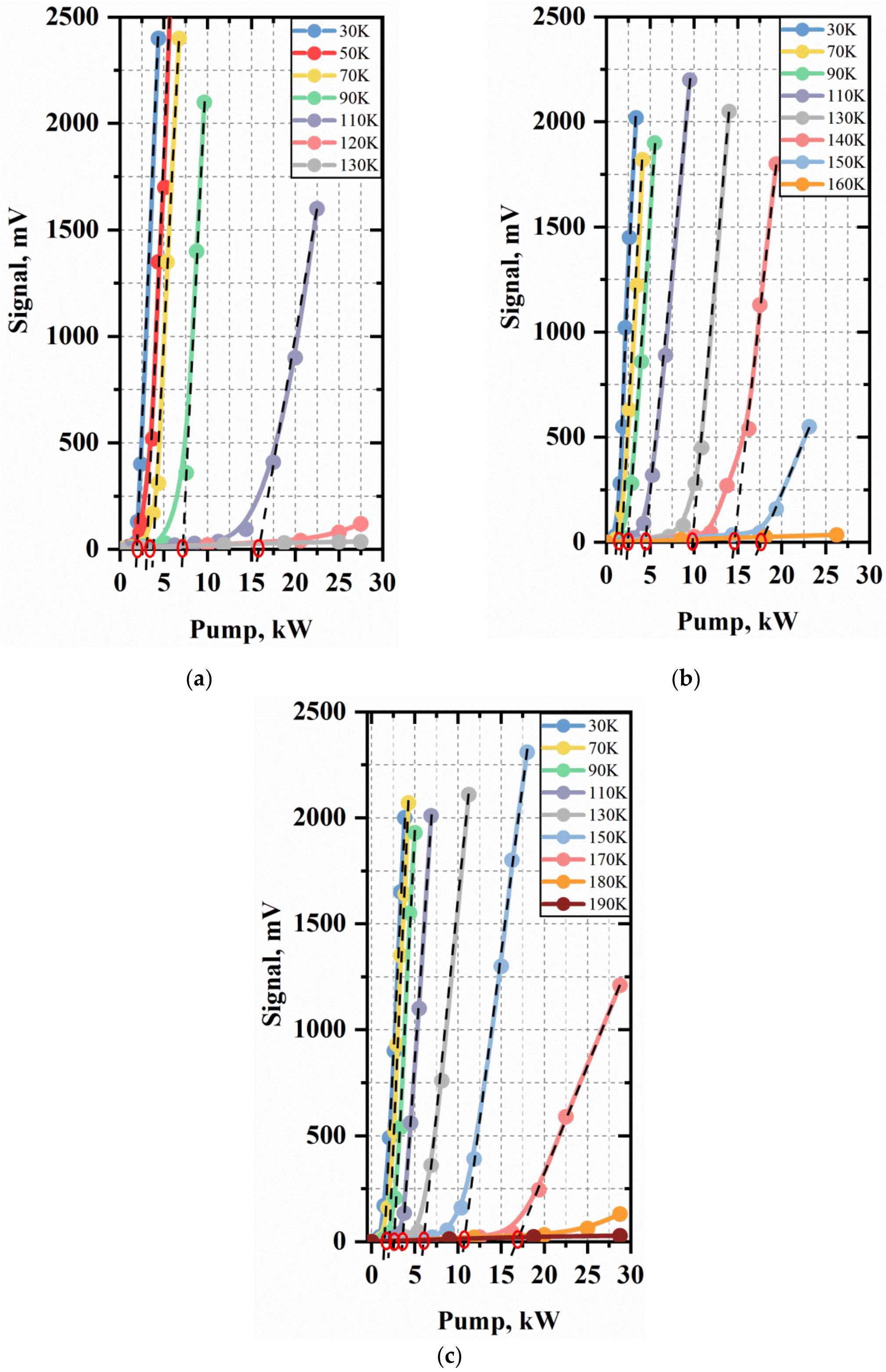

Figure S2. Dependence of PL signal intensity on pumping power at different temperatures for structures with: (**a**) 3 QWs; (**b**) 5 QWs; (**c**) 10 QWs.

Threshold pumping intensity was determined from the measurements of PL signal vs pumping intensity and linear extrapolation of above threshold signal to zero. For all studied structures the slopes of the measured dependencies above the threshold decrease with temperature while threshold intensity increases. For structures with less QWs in the active region, the threshold pumping intensity is higher, which is accompanied by lower

signal in the same experimental conditions. At critical temperature threshold intensity becomes higher than maximum intensity of the pumping laser, as a result, at temperatures above critical no SE was obtained. Critical temperature was measured to be 120, 150 and 170 K for structures with three, five and ten QWs, correspondingly. Figure 3 shows the fitting of the experimental data.

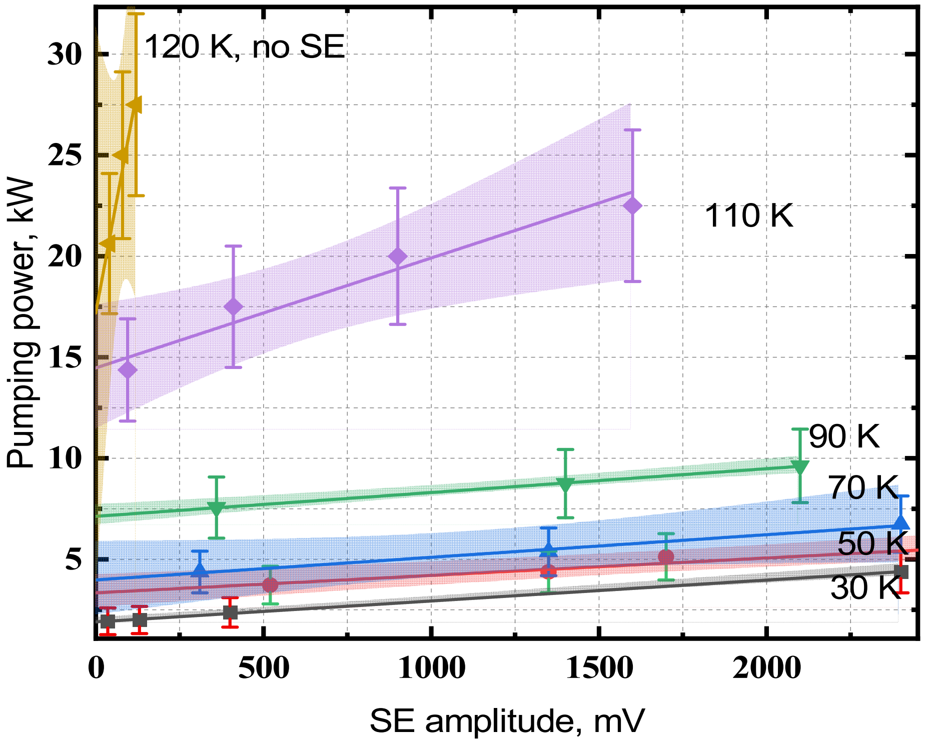

Figure S3. Pumping power versus stimulated emission intensity at different temperatures for structures with 3 QWs. Symbols correspond to experimental points with error, solid curves are the fitted curves, semitransparent areas denote 95% confidence intervals.
